# An infantile exogenous Cushing syndrome caused by topical steroid

**DOI:** 10.1186/1687-9856-2015-S1-P45

**Published:** 2015-04-28

**Authors:** Palinee Nantarakchaikul, Kachaporn Nimdet

**Affiliations:** 1 Suratthani Central Hospital, Suratthani, Thailand

## 

Corticosteroids are widely use for the treatment of various diseases. Long-term use of corticosteroids may lead to development of Cushing syndrome and hypothalamic pituitary adrenal axis suppression. However, iatrogenic Cushing syndrome from absorption of topical steroids to systemic circulation is less common comparing to parenteral or oral use. Infantile age group have greater risks for systemic side effects of the drugs because their skin has poorly developed barrier function and large surface area.

We reported a 5-month-old female child was admitted to our hospital with puffiness of face and excessive weight gain after 4 months old. She was born at term with an uneventful pregnancy with a birth weight of 3.4 kg. Her body weight was 8.2 kg (+2SDS), height was 63.5 cm (+0.2SDS). Physical examination showed her was moon face appearance, generalized obesity, hirsutism and buffalo hump. There were few erythema and weeping present at skin fold areas. Blood pressure was 105/68 mmHg (>95^th^ percentile). Past history revealed that the child had diaper dermatitis at 3.5 month of age. She was prescribed a topical combination drug containing nystatin, neomycin, gramicidin and triamcinolone acetonide from private pediatric clinic. Since then she was applying the drug 3-4 times daily on neck, axilla, diaper area and all skin fold areas. Totally drug usage was 30 g in 6 weeks. Investigations showed hemoglobin of 15 g/dL, white blood cell count 18,240/mm^3^, platelet count 471,000/mm^3^, blood glucose 74 mg/dL and normal electrolyte, renal and liver function. Ultrasonography of whole abdomen revealed no abnormality. Random PM cortisol was low (0.2 mcg/dL). Secondary adrenal insufficiency was suspected. 1-mcg ACTH stimulation test was done and the peak cortisol was 1.9 mcg/dL. Therefore, exogenous Cushing syndrome and secondary adrenal insufficiency due to overuse of topical combination drug which containing moderate potency steroid was diagnosed. She was promptly treatment by discontinued applying the drug and administered steroid during stress.

This report highlights the importance of prolonged use of moderate potency topical steroids can causes hypothalamic pituitary adrenal axis suppression and Cushing syndrome, especially during infantile period. Therefore, limiting the use of moderate- to high-potency topical steroids in children and provide information about potential adverse effects to the caregivers are crucial.

Written informed consent was obtained from the patient for publication of this abstract and any accompanying images. A copy of the written consent is available for review by the Editor of this journal.

